# Deciphering the gut-lung axis in non-small cell lung cancer: from immune microenvironment crosstalk to clinical translation

**DOI:** 10.3389/fonc.2026.1975256

**Published:** 2026-10-27

**Authors:** Changrong Sun, Fei Han, Wenhao Xu, Xinliang Wang, Xin Wang

**Affiliations:** 1 School of Clinical Medicine, Shandong Second Medical University, Weifang, Shandong, China; 2 Department of Respiratory and Critical Care Medicine, Central Hospital Affiliated to Shandong First Medical University, Shandong First Medical University & Shandong Academy of Medical Sciences, Jinan, Shandong, China

**Keywords:** gut-lung axis, immunotherapy resistance, metabolic reprogramming, microbiota, non-small cell lung cancer, tumor immune microenvironment

## Abstract

Immune checkpoint inhibitors (ICIs) targeting the PD-1/PD-L1 axis have established a new standard of care for non-small cell lung cancer (NSCLC); however, clinical efficacy is frequently limited by primary resistance, acquired (secondary) resistance, and immune-related adverse events. The heterogeneity of the tumor immune microenvironment (TIME) is a critical determinant of therapeutic response, a state effectively regulated by the gut microbiota via the gut-lung axis. This review systematically examines the mechanistic crosstalk between intestinal commensals and the pulmonary TIME. We first delineate how specific bacterial metabolites, such as short-chain fatty acids and indole-3-acetic acid, alongside bacterial-fungal trans-kingdom interactions, modulate innate and adaptive immune responses within the lung through epigenetic remodeling and metabolic signaling, and we map the discrete points at which these gut-derived signals converge on the intrinsic metabolic programmes of pulmonary tumor cells. Furthermore, we analyze the dual role of the microbiome as a predictive biomarker, discussing the evolution from simple taxonomic abundance profiling to complex ecological topology scores and the identification of therapeutic toxicity driven by molecular mimicry, and we examine whether pre-intervention alpha or beta diversity metrics are required for patient selection. Finally, we evaluate emerging therapeutic strategies, including next-generation probiotics, bacterial-derived extracellular vesicles, and combinational interventions that aim to overcome ICI resistance by restructuring the host-microbe ecosystem, with particular attention to acquired resistance to PD-1 blockade. We also critically appraise the barriers that currently impede clinical translation, including the inter-cohort non-reproducibility of taxonomic biomarkers, methodological heterogeneity between 16S amplicon and shotgun metagenomic profiling, and the safety and regulatory constraints on fecal microbiota transplantation in immunocompromised patients. This synthesis underscores the necessity of integrating microbiome profiling into standard oncological practice to facilitate precision immunotherapy.

## 1 Introduction

Non-small cell lung cancer (NSCLC) remains the leading cause of cancer-related mortality worldwide. While the advent of immune checkpoint inhibitors (ICIs) targeting the PD-1/PD-L1 axis has established a new standard of care, clinical reality is characterized by a significant dichotomy: a fraction of patients achieve durable remission, while the majority succumb to primary or acquired resistance or immune-related adverse events (irAEs). The therapeutic efficacy of ICIs is fundamentally dictated by the tumor immune microenvironment (TIME), specifically the magnitude of cytotoxic T cell infiltration and the metabolic fitness of effector cells. However, accumulating evidence indicates that the TIME is not an isolated local ecosystem but is dynamically regulated by systemic physiological factors, most notably the gut microbiota.

The gut-lung axis represents a bidirectional communication network wherein intestinal dysbiosis can remotely impair pulmonary immunity. Epidemiological data demonstrate that antibiotic-induced disruption of the gut flora significantly compromises the survival benefits of ICIs in patients with NSCLC. Beyond this phenomenological association, recent mechanistic studies have revealed that gut-derived metabolites, immune cells primed in mesenteric lymph nodes, and trans-kingdom interactions between bacteria and fungi serve as critical messengers modulating the antitumor response within the lung. Despite these advances, the translation of microbiome research into clinical oncology faces substantial hurdles, including the complexity of microbial ecology and the lack of standardized biomarkers.

Unlike previous reviews that predominantly focus on bacterial taxonomic abundance, this article integrates the latest findings on metabolic reprogramming, the gut mycobiome, and novel delivery systems such as extracellular vesicles (EVs). We aim to provide a comprehensive framework elucidating how the gut microbiome orchestrates the NSCLC immune microenvironment and to highlight actionable strategies for manipulating this axis to reverse immunotherapy resistance. To this end, Section 2 defines the intrinsic immune and metabolic landscape of NSCLC and identifies, node by node, the points at which gut-derived signals intersect with it; Section 3 dissects the systemic mechanisms of gut-lung communication, including the temporal kinetics of antibiotic-induced depletion of metabolite-producing strains; Section 4 evaluates microbiota-directed therapeutic strategies, with explicit attention to acquired resistance to PD-1 blockade; Section 5 assesses the predictive value and the limitations of microbiome-based biomarkers; and Section 6 appraises the barriers to clinical translation and summarizes ongoing trials.

## 2 The intrinsic landscape of NSCLC and its convergence with the gut-lung axis

The features summarized below are conventionally framed as tumor-intrinsic properties. However, an expanding body of evidence indicates that several of these programmes are also remote targets of gut-derived signals conveyed through the gut-lung axis. This section therefore describes the intrinsic immune and metabolic landscape of NSCLC and, at each node, identifies the specific points at which intestinal commensals and their metabolites have been shown to impinge upon it; the systemic mechanisms responsible are dissected in Section 3 (**Figure 1**).

**Figure 1:**
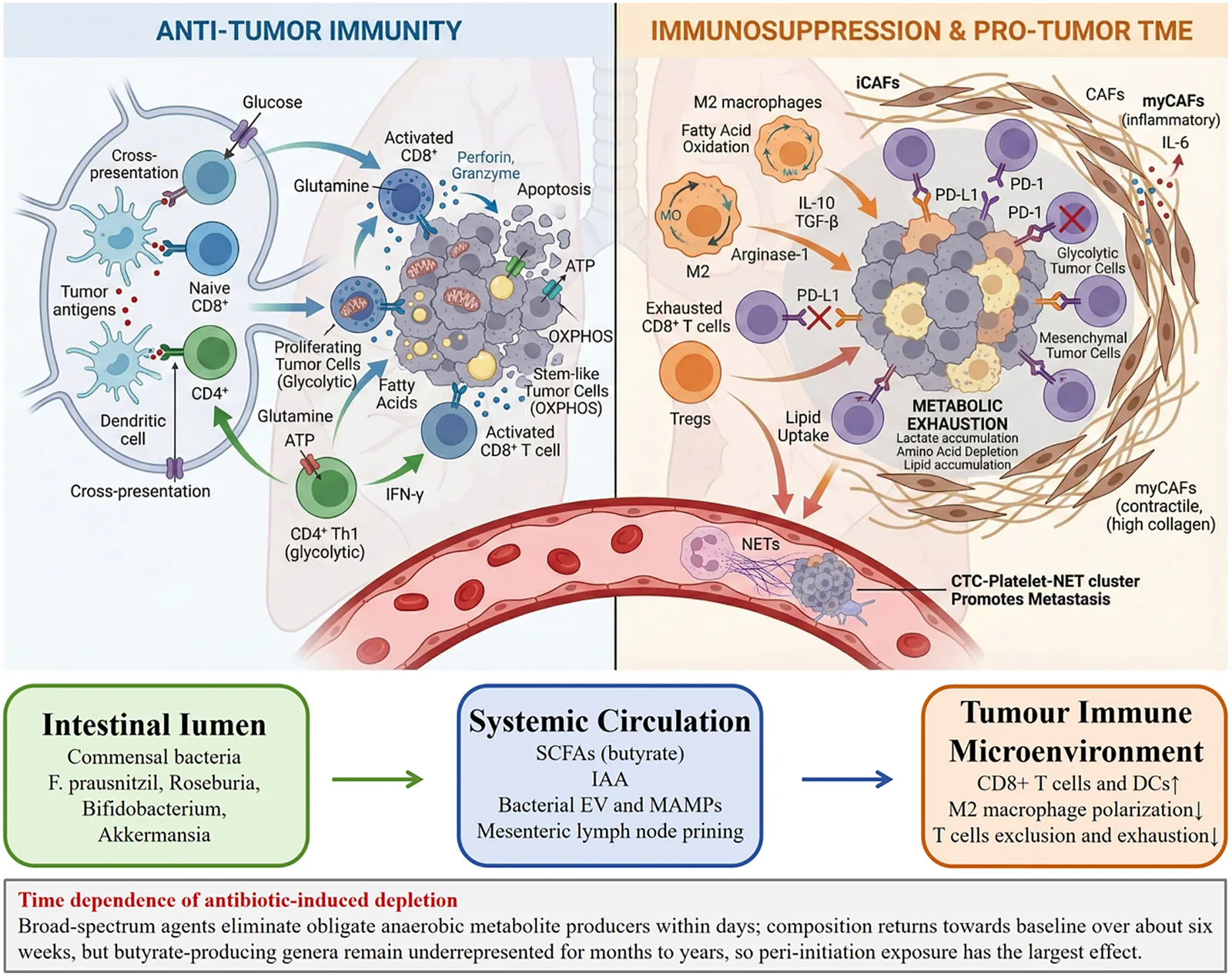
The landscape of immune heterogeneity and metabolic reprogramming in the lung cancermicroenvironment, together with its systemic gut-derived inputs. Anti-tumor immunity. The left panel depicts an effective immune surveillance state. Dendritic cells (DCs) uptake tumor antigens and cross-present them to naive T cells. Activated CD8+ cytotoxic T cells (CTLs) infiltrate the tumor core and induce apoptosis via the release of perforin and granzymes, supported by IFN-gamma secreted by CD4^+^ Th1 cells. Immunosuppression and pro-tumor reprogramming. The right panel illustrates the mechanisms of immune escape. Tumor cells express PD-L1 to bind PD-1 on T cells, leading to T cell exhaustion. Immunosuppressive cells, including M2 macrophages and Tregs, secrete inhibitory cytokines (IL-10, TGF-beta). CAFs form a dense collagen barrier and drive metabolic competition (glucose depletion and lactate accumulation), creating a hostile microenvironment that restricts T cell function. Metastatic niche formation. The bottom vessel section highlights the systemic risk where neutrophils release NETs to capture circulating tumor cells (CTCs), facilitating their survival and hematogenous metastasis. Gut-derived systemic inputs to the pulmonary tumor immune microenvironment. Commensal bacteria in the intestinal lumen generate metabolites, including short-chain fatty acids and indole derivatives, which enter the systemic circulation together with microbiota-primed immune cells. Butyrate acts as a histone deacetylase inhibitor in CD8^+^ T cells; indole-3-acetic acid (IAA) acts as an aryl hydrocarbon receptor (AhR) ligand that restrains the METTL3-STAT3 axis and blocks M2 macrophage polarization; dendritic cells and T cells primed in the mesenteric lymph node migrate through the thoracic duct to the lung, where they modulate the tumor immune microenvironment described above. Perturbation of this input by antibiotics removes the metabolite-producing strains and shifts the pulmonary microenvironment towards the immunosuppressive state described above.

### 2.1 Immune and stromal heterogeneity of the pulmonary tumor microenvironment

The lung cancer tumor immune microenvironment (TIME) is a dynamic ecosystem woven together by immune cells, stromal components, and their metabolic networks. Its high degree of heterogeneity and metabolic plasticity are core mechanisms leading to immune escape and therapeutic resistance. Recent multi-omics studies have primarily revealed the functional remodeling characteristics of lymphocyte subsets within the microenvironment. Specifically, CD4^+^ tissue-resident memory T cells (CD4^+^ TRMs) play a critical role in immune surveillance in non-small cell lung cancer (NSCLC). Research has found that CD4^+^ TRMs are intended to recruit type 1 conventional dendritic cells (cDC1) by secreting the chemokine XCL1, thereby initiating a specific anti-tumor immune cycle. However, tumor cells block this TRM-cDC1 axis by downregulating the key costimulatory molecule JAML on the surface of these T cells, resulting in the failure of immune priming (1). Simultaneously, tumor cells utilize extracellular vesicles (EVs) as vehicles for long-range immunosuppression. After being taken up by CD4^+^ T cells, EVs carrying PD-L1 induce cell cycle arrest at the G1/S phase. Targeted inhibition of lysine-specific demethylase 1 (LSD1) has been shown to effectively block this process and restore T cell proliferative vitality (2).

Beyond inter-patient differences, the NSCLC TIME is also heterogeneous within individual tumors. Immune cell composition, checkpoint expression and metabolic activity vary substantially between geographically distinct regions of the same lesion, so that immune-inflamed (“hot”), immune-excluded and immune-desert (“cold”) compartments coexist within a single tumor. This intratumoral heterogeneity is one of the principal reasons why a single baseline biopsy predicts ICI benefit only imperfectly, and it provides the rationale for the cold-to-hot conversion strategies discussed below (3).

In addition to cellular components, the metabolic reprogramming of stromal cells acts in concert with the physicochemical properties of the microenvironment to constitute a physical barrier limiting the immune response. Single-cell transcriptome sequencing has identified a population of senescence-associated fibroblasts (CAFs) characterized by p16^Ink4a+^ expression. These cells are not passive bystanders but actively promote fatty acid uptake and utilization in tumor cells via paracrine signaling, inducing tumor cells to enter a highly plastic state that drives malignant progression (4). Furthermore, in NSCLC tissues with hyperactivated Wnt signaling pathways, the extremely high glycolytic activity of tumor cells leads to excessive lactate accumulation. This acidic microenvironment directly results in T cell exclusion, and utilizing supramolecular strategies to neutralize this acidity has been proven effective in reversing T cell exclusion and rebuilding anti-tumor immunity (5).

Beyond these populations, regulatory B cells that display the inhibitory ligand VISTA and adopt a metabolically activated, highly polyfunctional phenotype accumulate in early-stage NSCLC and are associated with tumor recurrence, further broadening the repertoire of immunosuppressive cells that constrain effector T cell function (23).

Notably, metastatic sites of lung cancer often form unique immune-privileged microenvironments, a phenomenon particularly prominent in brain metastasis models. Upon reaching the brain, lung cancer cells secrete Lipocalin-2 (LCN2), which binds to the SLC22A17 receptor on astrocytes, activating the JAK2/STAT3 signaling pathway. This induces astrocytes to secrete inflammatory chemokines and recruit macrophages, thereby actively establishing a microenvironment supportive of tumor growth (6). Concurrently, neutrophils in brain metastases specifically protect LOX^+^ metastasis-initiating cells by releasing neutrophil extracellular traps (NETs), promoting their epithelial-mesenchymal transition and subsequent colonization (7).

### 2.2 Carbon source plasticity and environmental adaptation

While the Warburg effect is a hallmark of NSCLC, recent evidence reveals a highly plastic carbon metabolism driven by environmental pressures (**Figure 2**). Tobacco smoke exposure, a primary risk factor, directly rewires glycolysis by inducing the succinylation of GAPDH at Lys251, locking cells in a high-glycolytic state to support anabolic growth (8). Beyond glucose, tumors flexibly utilize alternative fuels; for instance, the activation of the polyol pathway allows NSCLC cells to metabolize fructose under glucose deprivation, driving malignancy via ALDOB-mediated signaling (9). Crucially, metabolic byproducts function as signaling molecules rather than mere waste. Acetate acts as an epigenetic modulator by acetylating the promoter of CD274 (PD-L1), directly linking metabolism to immune checkpoint evasion (10). Furthermore, emerging environmental pollutants like 6PPD from tire wear particles disrupt the TCA cycle through ARG2-mediated arginine metabolism (11). This metabolic flexibility is supported by redox adaptation, where the N4-acetylcytidine (ac4C) modification of lncRNA LINC02802 maintains the mitochondrial NAD+/NADH balance (12), and non-canonical antioxidant pathways involving GAPDH prevent the accumulation of toxic advanced glycation end-products (AGEs) (13).

**Figure 2:**
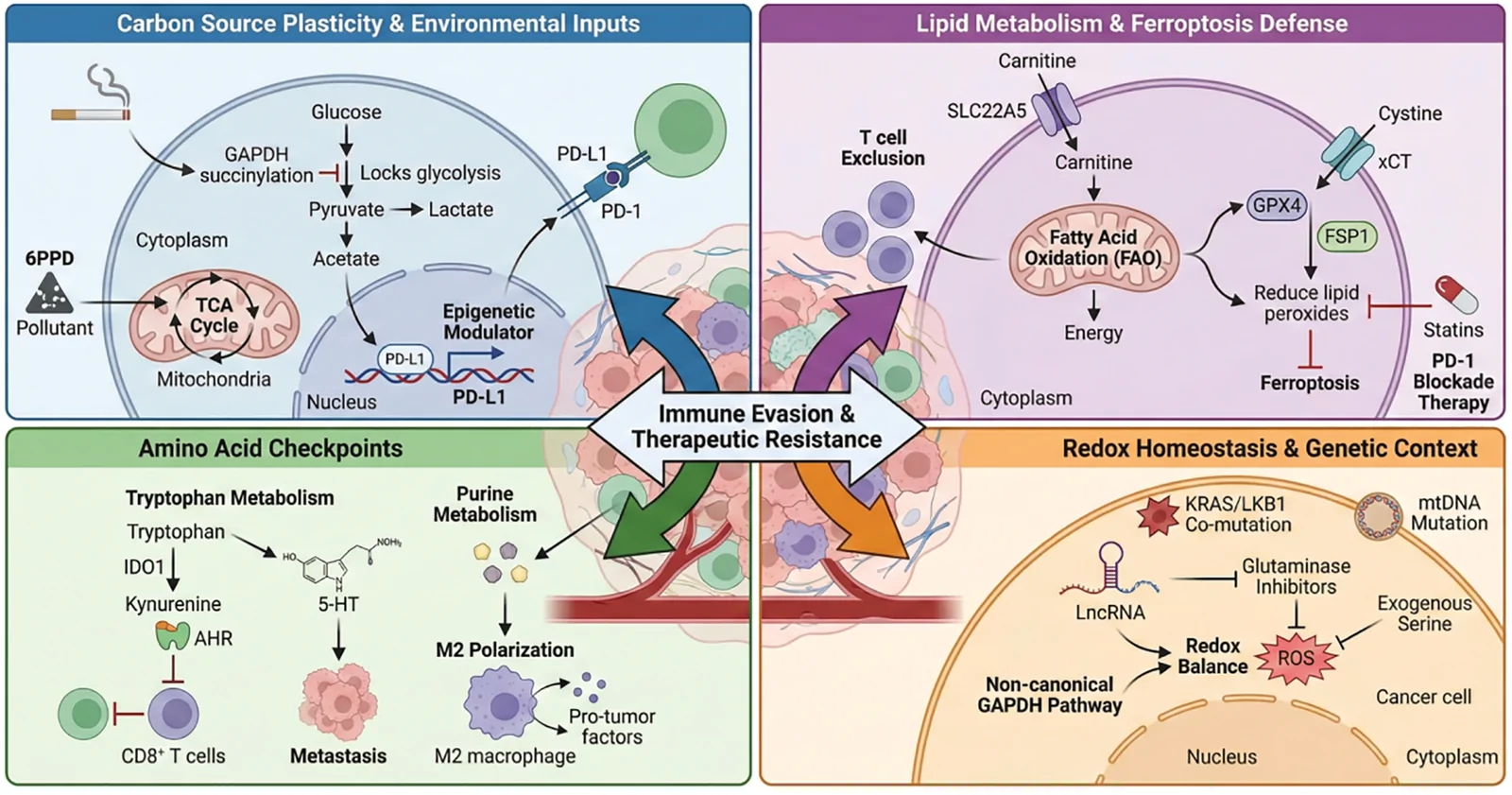
Intrinsic metabolic reprogramming of NSCLC cells: the engine of immune evasion and therapeutic resistance. Carbon source plasticity and environmental inputs. Environmental carcinogens, such as cigarette smoke, induce GAPDH succinylation to lock cells in a glycolytic state, while the pollutant 6PPD disrupts the TCA cycle. Metabolic byproducts, particularly acetate, function as epigenetic modulators to directly upregulate PD-L1 expression, facilitating immune escape. Lipid metabolism and ferroptosis defense. Upregulated SLC22A5 mediates carnitine uptake to fuel fatty acid oxidation, creating a lipid-metabolic barrier that drives T cell exclusion. Concurrently, NSCLC cells leverage the xCT/GPX4/FSP1 axis to neutralize lipid peroxides and evade ferroptosis. Targeting this pathway with statins can sensitize tumors to PD-1 blockade. Amino acid checkpoints. Tryptophan metabolism exerts dual pro-tumorigenic effects: the IDO1-kynurenine-AHR axis inhibits CD8^+^ T cell cytotoxicity, while serotonin (5-HT) signaling promotes metastasis. Additionally, aberrant purine metabolism drives the polarization of tumor-associated macrophages towards an immunosuppressive M2 phenotype. Redox homeostasis and genetic context. Specific genetic landscapes, including KRAS/LKB1 co-mutations and mitochondrial DNA mutations, create distinct metabolic dependencies, such as hypersensitivity to glutaminase inhibitors or exogenous serine deprivation. lncRNAs and non-canonical GAPDH pathways are critical for maintaining redox balance and neutralizing ROS.

### 2.3 Lipid metabolism, ferroptosis, and organelle stress

Lipid metabolic reprogramming in NSCLC extends far beyond SREBP1-driven lipogenesis (14). It involves a sophisticated balance between storage, oxidation, and ferroptosis defense. The aberrantly expressed lipid droplet protein CIDEC protects tumor cells from lipotoxicity and promotes survival (15), while albumin-bound statins targeting cholesterol-rich lipid rafts have been shown to sensitize tumors to PD-1 blockade (16). Mitochondrial fatty acid oxidation (FAO) acts as a metabolic barrier to immunotherapy; the carnitine transporter SLC22A5 facilitates carnitine influx to fuel FAO, driving a T-cell exclusion phenotype (17). Conversely, manipulating lipid peroxidation offers a therapeutic window. Targeting the cystine/glutamate antiporter xCT (SLC7A11) triggers ferroptosis (18), a process further regulated by hydrogen sulfide (H2S) signaling and the FSP1-CoQ10 axis (19, 20). However, tumors evolve resistance mechanisms, including the use of methionine-SAM metabolism to support ubiquinone synthesis and thereby modulate lipid peroxide accumulation, and remodeling of phospholipid composition through ACSL4 to alter sensitivity to ferroptosis-inducing agents (21, 22).

### 2.4 Amino acid checkpoints as immunosuppressive hubs

Tumor cells aggressively scavenge amino acids, establishing metabolic checkpoints that starve immune effector cells. Tryptophan metabolism is particularly critical; the methylation of TFAP2A by PRMT3 upregulates IDO1, producing kynurenine which activates the Aryl Hydrocarbon Receptor (AHR) to suppress CD8^+^ T cells and promote radioresistance (24). Simultaneously, a non-canonical serotonin (5-HT) pathway driven by the SNRPG/WT1/CDK14 axis enhances metastatic colonization (26). Glutamine and serine metabolism are equally pivotal; tumors utilize glutamine not only for biosynthesis but also to modulate the epigenetic landscape via alpha-ketoglutarate (27). In specific genetic contexts, such as tumors with mitochondrial DNA (mtDNA) mutations, a respiration defect creates a strict dependency on exogenous serine (28). Furthermore, the catabolism of branched-chain amino acids (BCAAs) by BCAT1 fuels proliferation (29), while limiting purine metabolism in macrophages can reverse their immunosuppressive M2 polarization (30).

### 2.5 Metabolic heterogeneity, therapeutic vulnerabilities, and convergence of gut-derived signals

The metabolic landscape of NSCLC is highly heterogeneous, dictated by oncogenic drivers and oxygen availability. KRAS/LKB1 co-mutated tumors exhibit unique dependencies on the KEAP1-NRF2 pathway to manage oxidative stress, creating specific vulnerabilities to glutaminase inhibition (31). Hypoxia further complicates this landscape by stabilizing HIF-1α to drive glycolytic enzymes and reshaping the extracellular matrix via lactate-induced signaling (32–35). Recent strategies have exploited these vulnerabilities: inhibiting mitochondrial lipoylation overcomes radioresistance, and targeting NAMPT disrupts the NAD+ salvage pathway crucial for tumor survival (36, 37).

An additional layer of heterogeneity arises from genomic instability, because LINE-1 retrotransposon activity promotes tumorigenicity and exacerbates progression by stimulating metabolic reprogramming in NSCLC, indicating that the metabolic landscape is continuously reshaped by the tumor’s own mutational history (25).

Importantly, several of the metabolic nodes described above are not autonomous tumor-cell programmes; they are directly accessible to gut-derived signals, and this convergence provides the mechanistic bridge between the tumor-intrinsic landscape summarized here and the gut-lung axis dissected in Section 3. Four parallels are particularly well documented. First, with respect to carbon and epigenetic metabolism, the microbial metabolite butyrate acts on tumor cells through histone deacetylase inhibition: in lung adenocarcinoma, intratumoral butyrate inhibits HDAC2, increases H3K27 acetylation at the H19 promoter, and drives MMP15 secretion and M2 macrophage polarization, whereas higher butyrate concentrations suppress tumor-cell PD-L1 through acetylation of STAT1. The same metabolite therefore impinges directly on the acetate-PD-L1 axis and on the glycolytic-epigenetic coupling described above, with the direction of the effect determined by concentration and compartment (68). Second, the tryptophan-indole node is shared between host and microbe: whereas tumor-intrinsic PRMT3-IDO1 signaling generates kynurenine as an AHR ligand (24), the bacterial indole derivative trans-3-indoleacrylic acid signals through the same receptor to upregulate ALDH1A3 and, via FSP1-dependent coenzyme Q10 reduction, to inhibit ferroptosis in tumor cells, conceptually linking the amino acid checkpoint described in Section 2.4 to the lipid-peroxidation defense described in Section 2.3 (69). Third, indole-3-acetic acid produced by Bifidobacterium animalis restrains the METTL3-STAT3 axis in lung cancer cells, an m6A-dependent programme that converges on the same macrophage polarization node as the tumor-intrinsic purine and tryptophan pathways (40). Fourth, indirect gut-to-tumor signaling occurs through loss of intestinal barrier integrity and the resulting systemic inflammatory tone, which has been linked to a pro-inflammatory, tumor-promoting pulmonary microenvironment (47). Collectively, these observations indicate that the tumor-intrinsic metabolic landscape is best regarded as the downstream integration point of both oncogenic and microbial inputs, which is why metabolic and microbiome-directed interventions are expected to be synergistic rather than redundant (38, 39).

## 3 Systemic regulation mechanisms of the lung cancer immune microenvironment via the gut-lung axis

Despite the significant anatomical distance, the gut microbiota exerts profound systemic regulation on the lung cancer immune microenvironment by constructing a sophisticated Gut-Lung Axis communication network (**Figure 3**), utilizing mechanisms such as metabolite diffusion, cell migration, and physical translocation. The most clearly defined mechanism currently is the entry of gut-derived bioactive metabolites into the lung via blood circulation, where they induce epigenetic remodeling of tumor and immune cells. For instance, addressing the immune suppression caused by the common overexpression of METTL3 in lung cancer, the metabolite indole-3-acetic acid (IAA) produced by Bifidobacterium animalis acts as a ligand for the aryl hydrocarbon receptor (AHR). IAA specifically inhibits METTL3 expression, subsequently reducing the m^6^A modification levels and stability of STAT3 mRNA, successfully blocking STAT3-mediated M2 macrophage polarization (40).

**Figure 3:**
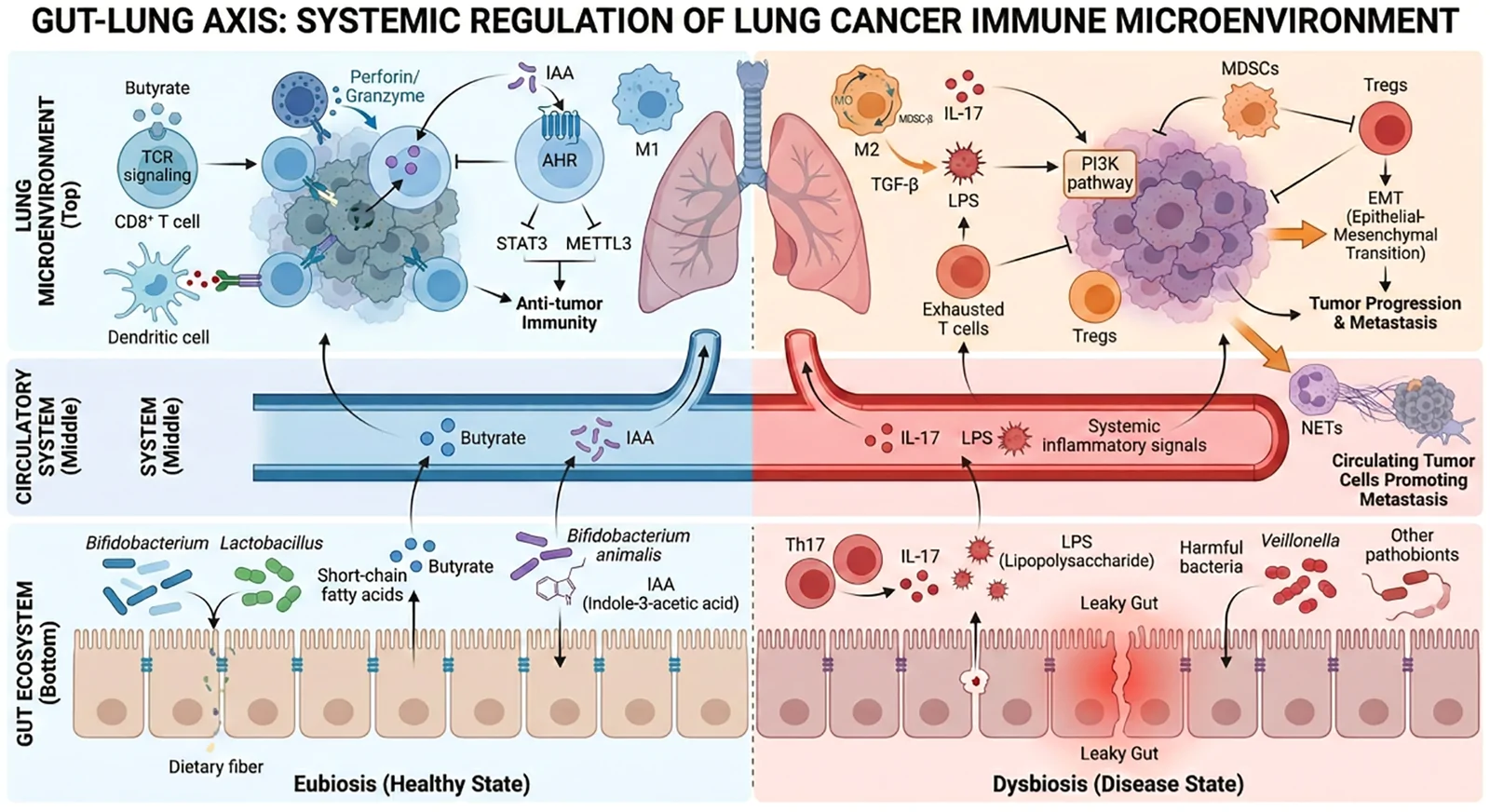
Systemic regulation of the lung cancer immune microenvironment via the gut-lung axis. Eubiosis and anti-tumor regulation (left). Under healthy conditions (eubiosis), beneficial commensals (e.g., Bifidobacterium animalis) produce bioactive metabolites. IAA acts as a ligand for the aryl hydrocarbon receptor (AHR), suppressing METTL3 expression and inhibiting the STAT3 signaling pathway, thereby blocking M2 macrophage polarization. Butyrate functions as a histone deacetylase inhibitor (HDACi) to increase histone acetylation at Pdcd1 and Cd28 loci, enhancing TCR signaling sensitivity and cytotoxicity in CD8^+^ T cells. Dysbiosis and pro-tumor progression (right). Gut dysbiosis (e.g., abundance of Veillonella) leads to a compromised intestinal barrier. Systemic inflammation: translocation of LPS and pathogenic bacteria, along with elevated IL-17, triggers systemic inflammation. Microenvironmental remodeling: these signals reach the lung to activate the PI3K/Akt pathway, promote epithelial-mesenchymal transition (EMT), and recruit suppressor cells (MDSCs, Tregs), ultimately accelerating tumor progression and metastasis. Antibiotic exposure acts on this axis in a time-dependent manner: broad-spectrum agents eliminate obligate anaerobic metabolite producers within days, and functional recovery of butyrate-producing taxa lags behind compositional recovery for weeks to months, so that the interval between antibiotic exposure and immunotherapy initiation determines the magnitude of the effect.

Regarding the regulation of T cell function, short-chain fatty acids produced by gut microbial metabolism have demonstrated remarkable epigenetic regulatory capabilities. Butyrate, acting as a natural histone deacetylase inhibitor, significantly upregulates H3K27ac acetylation levels in the promoter regions of the Pdcd1 and Cd28 genes in CD8^+^ T cells. This remodeling enhances the efficiency of downstream TCR signaling, directly boosting the cytotoxic function of T cells and their responsiveness to PD-1 blockade therapy (41); consistent with this, integrated microbiota-metabolite analyses in NSCLC have linked gut short-chain fatty acid profiles to the efficacy of combined chemotherapy and immunotherapy (54). Additionally, microbial metabolites play roles in post-translational modifications and cytoskeletal regulation; for example, mannose inhibits the transcription of the inflammatory factor IL-8 by targeting the OGT/hnRNP R axis (42), while Urolithin A limits the physical invasive capability of cancer cells by activating autophagy pathways to degrade the cytoskeletal protein TMSB10 (43).

The vulnerability of this metabolite-dependent crosstalk is defined by the kinetics of antibiotic-induced depletion, which explains why the timing of antibiotic exposure relative to immunotherapy initiation is so consequential. Because butyrate producers such as Faecalibacterium prausnitzii, Roseburia and members of the Lachnospiraceae and Ruminococcaceae are obligate anaerobes, they are among the taxa most rapidly and most durably eliminated by broad-spectrum agents. In healthy adults given a four-day course of meropenem, gentamicin and vancomycin, butyrate-producing species and Bifidobacterium spp. were depleted within days; although the community returned to near-baseline composition within approximately six weeks, nine common species that were present before treatment remained undetectable in most subjects at day 180, indicating that functional recovery lags behind compositional recovery (65). Controlled comparisons across antibiotic classes confirm this pattern: after clindamycin, ciprofloxacin, amoxicillin or minocycline, butyrate-producing genera including Faecalibacterium, Roseburia, Coprococcus, Subdoligranulum and uncultured Lachnospiraceae remained strongly underrepresented for months, with predicted butyrate-synthesis pathways correspondingly reduced (66); and at the extreme, a seven-day clindamycin course produced disturbances that persisted throughout a two-year follow-up, with the Bacteroides community never returning to its original composition (67). Clinically, this temporal window is reflected in the observation that the adverse impact of antibiotics on ICI outcome is concentrated in a narrow peri-initiation period, whereas antibiotics administered concurrently with checkpoint blockade were not associated with worse survival in the same cohort, indicating that the window rather than the antibiotic itself is the critical variable (51). Because the same obligate anaerobes that produce butyrate also generate indole derivatives, a single antibiotic course can silence both the epigenetic and the AHR-ligand arms of the axis simultaneously, which provides a mechanistic explanation for the disproportionate effect of peri-initiation antibiotic exposure.

Beyond the remote delivery of metabolic molecules, cross-organ migration of immune cells and systemic metabolic competition are also important modes of action for the Gut-Lung Axis. Commensal bacteria such as Segmented Filamentous Bacteria (SFB) can induce the production of Th17 cells. These cells possess specific homing capabilities, allowing them to migrate to lung cancer tissues and transdifferentiate into Th1-like cells to enhance anti-tumor effects (44). However, nutritional competition between the gut microbiota and the host may also bring negative impacts. For example, asparaginase produced by Bacteroides species can deplete asparagine in the systemic circulation, and this amino acid deprivation impairs the metabolic adaptability and proliferative capacity of CD8^+^ T cells via the SLC1A5 transporter (45).

Finally, the physical translocation of microbial entities and alterations in the local microecology directly reshape the local microenvironment of lung cancer. Recent spatial metabolomics evidence reveals that Akkermansia muciniphila not only colonizes the gut but can also penetrate barriers to migrate inside lung cancer tissues, exerting anti-cancer effects by remodeling local glucose and glutamate metabolic networks (46). Conversely, if the local lung environment is enriched with oral-derived anaerobic bacteria such as Veillonella, it can induce a pro-inflammatory microenvironment by upregulating IL-17 and the PI3K pathway, accelerating tumor progression (47). Based on these mechanisms, natural products such as ginseng polysaccharides, the green tea extract EGCG, and Aconite (Fuzi) improve the lung cancer immune microenvironment precisely by regulating microbial structure and correcting metabolic disorder (48–50, 53).

## 4 Gut microbiota-mediated therapeutic regulation in non-small cell lung cancer

The increasing clarity of the Gut-Lung Axis mechanisms has facilitated the transition of microbiota from a prognostic biomarker to a tangible therapeutic target. Current strategies primarily focus on remodeling the dysbiotic gut ecosystem to overcome resistance to immune checkpoint inhibitors (ICIs), alleviate chemotherapy-induced toxicity, and correct systemic metabolic deficits such as cachexia.

### 4.1 Reshaping microbiota to potentiate immunotherapy: from primary to acquired resistance

Clinical evidence strongly suggests that the baseline state of the gut microbiota dictates the therapeutic threshold of ICIs. Retrospective analyses have confirmed that the use of antibiotics (ATB) within 30 days prior to or during ICI initiation induces a state of pathological dysbiosis, characterized by a reduction in diversity and the depletion of butyrate-producing bacteria. This antibiotic-induced disruption is significantly associated with shortened progression-free survival (PFS) and overall survival (OS) in patients with advanced NSCLC (51).

To reverse this resistance, Fecal Microbiota Transplantation (FMT) has emerged as a direct intervention strategy. Seminal studies demonstrated that transplanting fecal microbiota from ICI-responsive patients into germ-free or antibiotic-treated mice successfully reinstated the antitumor efficacy of PD-1 blockade; oral supplementation with Akkermansia muciniphila after transplantation of non-responder feces restored the efficacy of checkpoint blockade in an interleukin-12-dependent manner by increasing the recruitment of CCR9^+^CXCR3^+^CD4^+^ T lymphocytes into the tumor bed (64).

A critical distinction must be drawn between primary (*de novo*) and acquired (secondary) resistance, because the two populations differ in tumor biology, in the microbial alterations that accompany progression, and therefore in the expected benefit from microbiome-directed intervention. Most of the early FMT experience was generated in patients who had never benefited from PD-1 blockade, or in treatment-naive patients in whom FMT was administered before or with the first line of therapy, that is, as a strategy to prevent rather than to reverse resistance; the largest phase 2 and randomised trials conducted to date, including FMT-LUMINate and the PERFORM trial, adopted precisely this first-line design (38, 39). Acquired resistance, defined as progression after an initial objective response or prolonged disease control, now represents the numerically dominant clinical challenge in NSCLC and is mechanistically distinct, because the tumour has already been edited by immune pressure and has often acquired additional genetic and epigenetic alterations.

Evidence specifically addressing secondary resistance has only recently emerged, and it is derived from two prospective single-arm studies in advanced NSCLC. In the first, seven patients whose disease had progressed after an initial response to PD-1/PD-L1 blockade received oral faecal microbiota capsules from healthy donors together with re-challenge with a PD-1 inhibitor. The regimen was well tolerated, with only grade 1 FMT-related adverse events, but activity was modest: one partial response (objective response rate 14.3%), one stable disease (disease control rate 28.6%), median progression-free survival 1.5 months and median overall survival 12.1 months. Microbiome sequencing showed that alpha diversity increased in the responding patient and decreased in non-responders, indicating that the direction of the ecological shift, rather than the baseline state, tracked with outcome (70). In the larger second study, 27 patients with advanced NSCLC and documented secondary resistance to PD-1 blockade received oral faecal microbiota capsules in combination with PD-1 inhibitor re-challenge and chemotherapy. The objective response rate was 29.6% (8/27) with a disease control rate of 63.0%, median progression-free survival was 6.8 months and median overall survival was 11.1 months; responders displayed significantly increased gut microbial diversity together with enrichment of characteristic taxa, increased intratumoral CD8^+^ T cell infiltration and a reduced regulatory T cell fraction. Importantly, this study identified two modifiable negative prognostic factors in this specific sub-population: concomitant proton pump inhibitor use (HR 12.67, 95% CI 3.10-51.80, P < 0.001) and corticosteroid administration during FMT (HR 4.98, 95% CI 1.39-17.78, P = 0.013) (71).

Three conclusions follow from these data. First, FMT is feasible and acceptably tolerated in patients with acquired resistance, but the magnitude of benefit is substantially smaller than in the treatment-naive setting (objective response rates of approximately 15-30% versus 75-80% in first-line NSCLC), indicating that reversing an established resistance programme is considerably harder than preventing it (38, 70, 71). Second, the microbiome alterations that characterise secondary resistance are not a simple mirror image of those in primary resistance: whereas responders in first-line trials are distinguished by the loss of specific deleterious species, responders during re-challenge are distinguished by an increase in overall diversity after the intervention, suggesting that restoration of ecological breadth, rather than engraftment of a single beneficial taxon, may be the operative mechanism (38, 70, 71). Third, the identification of proton pump inhibitors and corticosteroids as dominant negative predictors implies that review of concomitant medication is at least as important as donor selection when microbiome-directed therapy is planned, and that these variables should be recorded prospectively in future trials (71).

Beyond bacteria, recent multi-cohort meta-analyses have expanded the therapeutic horizon to the gut mycobiome. It was found that stronger bacterial-fungal interactions are characteristic of ICI responders. Specifically, an increased abundance of Aspergillus pseudonomiae is associated with favorable prognosis in NSCLC, whereas deleterious fungi like Debaryomyces hansenii may impair therapeutic efficacy by reducing CD8^+^ T cell infiltration. This underscores the necessity of maintaining a balanced trans-kingdom ecosystem for optimal immunotherapy outcomes (52).

Furthermore, precision supplementation with specific probiotics offers a more controllable avenue. Supplementation with Clostridium butyricum (MIYAIRI 588, CBM588) has been shown to synergise with ICIs in lung cancer: in patients with advanced or recurrent NSCLC receiving checkpoint blockade, CBM588 therapy restored the efficacy that had been attenuated in those taking proton pump inhibitors, and was accompanied by enrichment of butyrate-producing taxa (80). More recent approaches involve engineering smart probiotics that accumulate in the tumour microenvironment and release immunomodulatory payloads locally, thereby decoupling efficacy from systemic toxicity (81).

### 4.2 Mitigating treatment-related toxicity and cachexia

The therapeutic potential of the microbiota extends to managing the systemic complications of lung cancer treatment. Cancer cachexia, a metabolic wasting syndrome prevalent in advanced NSCLC, severely limits patients’ tolerance to therapy. Multi-omics analysis has revealed that cachectic patients exhibit a distinct gut microbiome signature, characterized by the depletion of Lactobacillus gasseri and Prevotella copri. Functionally, this dysbiosis leads to a deficit in serum branched-chain amino acids (BCAAs) and 3-oxocholic acid, while enriching lipopolysaccharide (LPS) biosynthesis pathways that drive systemic inflammation (55).

Targeted dietary interventions or prebiotic supplementation aimed at restoring these specific taxa can correct metabolic derailment. Furthermore, regulating the gut-lung axis has shown promise in mitigating radiation-induced pneumonitis and chemotherapy-induced mucositis. By reinforcing the intestinal barrier function and reducing the translocation of inflammatory bacterial components, a balanced microbiota protects normal tissues from off-target damage caused by cytotoxic therapies (50, 62).

Collectively, these findings advocate for a paradigm shift in NSCLC management: moving from a tumor-centric approach to a holistic strategy that integrates gut microbiome modulation as a standard adjuvant to immunotherapy and supportive care, an approach now supported by prospective interventional evidence in NSCLC and by defined next-generation strains with demonstrated capacity to boost checkpoint blockade efficacy (38, 57).

## 5 The significance of gut microbiota in predicting non-small cell lung cancer outcomes

While therapeutic modulation remains a promising frontier, the immediate clinical utility of the gut microbiota lies in its potential as a non-invasive biomarker. Recent advancements have moved beyond simple taxonomic enumerations to complex ecological scoring systems and dynamic monitoring, providing robust predictive models for diagnosis, therapeutic efficacy, and toxicity in NSCLC.

### 5.1 Diagnostic signatures and early detection

The gut microbiota undergoes significant dysbiosis even in the early stages of lung cancer, offering a window for early screening. Large-scale cohort studies have identified specific microbial signatures (e.g., reduced Butyrivibrio and Roseburia) that distinguish early-stage NSCLC patients from healthy controls with high sensitivity, correlating strongly with classical serum tumor markers (59, 60).

### 5.2 Predicting immunotherapy efficacy: from taxa to ecological scores

The prediction of immune checkpoint inhibitor (ICI) response has evolved from identifying single beneficial taxa to evaluating the ecological topology and dynamic changes of the gut. A pivotal 2024 study introduced the TOPOSCORE, a unidimensional dysbiosis score based on bacterial co-occurrence networks, which successfully stratified patients into survival risk groups (61). Orthogonal readouts can complement such taxonomic models: integrated serum metabolomic profiling has identified amino acid and glycolytic signatures that act as modulators of immunotherapy response in NSCLC and may be combined with microbial features in multivariable prediction (56).

Crucially, in the specific context of locally advanced NSCLC treated with concurrent chemoradiotherapy (cCRT) followed by consolidation immunotherapy, longitudinal monitoring reveals that the dynamics of Akkermansia muciniphila are more predictive than baseline levels alone. Patients who exhibited a significant increase in Akkermansia abundance during the course of treatment achieved significantly longer progression-free survival (PFS) and overall survival (OS). This suggests that the microbiome’s adaptability to therapeutic stress is a key determinant of long-term clinical benefit (62).

A practical question follows directly from these observations: are specific baseline alpha or beta diversity metrics required before a microbiome-targeted intervention such as FMT can be expected to deliver predictive or therapeutic value? Present evidence does not support the use of a fixed pre-intervention diversity threshold, and no such threshold has been prospectively validated. The most comprehensive analysis of the determinants of strain dynamics after FMT identified the recipient’s pre-transplant species richness and the compositional dissimilarity between donor and recipient communities as the dominant predictors of post-transplant strain turnover, rather than baseline diversity indices considered in isolation; notably, lower pre-transplant richness predicted greater strain replacement rather than failure of engraftment (72). Consistent with this, in the phase 2 FMT-LUMINate trial the responders developed a distinct post-transplant community independently of acquired donor-recipient similarity or strain-level engraftment, indicating that the extent of overall engraftment is not the operative variable (38). In patients with acquired resistance to PD-1 blockade, baseline alpha diversity did not separate eventual responders from non-responders, whereas the direction of the diversity trajectory after intervention did (71). Donor characteristics appear to be at least as influential as recipient ecology: in the phase 1 PERFORM trial, donor-derived expansion of Segatella copri and enrichment of pro-inflammatory microbial enzymes in recipients were associated with grade 3 immune-related adverse events, and toxicity clustered among recipients of donors enriched in Prevotella species (38, 39). Taken together, these data argue that the pre-intervention variables worth measuring are not a single diversity index but a panel comprising pre-FMT species richness, donor-recipient community dissimilarity, the abundance of specific deleterious taxa that are targets for elimination, donor functional profile, and modifiable pharmacological exposures such as proton pump inhibitors and corticosteroids (71, 72). Baseline diversity metrics nonetheless remain valuable as descriptors of cohort-level dysbiosis and as covariates in multivariable models, and their standardized reporting should be encouraged.

### 5.3 Toxicity prediction and molecular mimicry

Predicting immune-related adverse events (irAEs) and treatment-related pneumonitis is as crucial as predicting efficacy. Recent multi-omics integration has revealed that molecular mimicry between gut microbial antigens and host tissues drives long-term toxicity (63).

Furthermore, for patients undergoing combined chemoradiotherapy and immunotherapy, gut dysbiosis serves as an early warning signal for severe lung toxicity. Research indicates that patients developing ≥ Grade 2 pneumonitis exhibit significantly lower alpha-diversity and distinct community shifts characterized by the enrichment of pathogenic taxa. This toxicity-associated dysbiosis likely exacerbates systemic inflammation, lowering the threshold for radiation-induced lung injury (62). Alpha diversity therefore retains value as a toxicity-oriented metric even though it is insufficient as a selection criterion for FMT, and these two applications should be distinguished explicitly when predictive models are designed.

## 6 Barriers to clinical translation and ongoing clinical trials

Despite the mechanistic coherence of the gut-lung axis described above, the translation of microbiome science into NSCLC practice is constrained by a set of interlocking problems that any clinical programme must confront. We address these in sequence - reproducibility, methodology, and safety and regulation - and then summarise the interventional trials of microbial therapeutics that have been registered in NSCLC and set out the specific roadblocks that must be cleared before a phase III trial can be mounted.

### 6.1 Limited cross-cohort reproducibility of microbiome-response associations

The first obstacle is the limited cross-cohort reproducibility of taxonomic biomarkers. In a meta-analysis of four shotgun metagenomic melanoma cohorts, samples clustered by study rather than by response, a separation between responders and non-responders was evident in only one dataset, and a classifier built on the pooled data performed poorly (area under the curve 0.625, 95% CI 0.348-0.899) (74). Similarly, a multi-cohort analysis of immune checkpoint blockade found that species-level signatures did not transfer between cohorts, and that reproducible performance across cancer types and countries was achieved only at strain resolution and only when the training and validation cohorts used concordant treatment regimens (58). Individual taxa illustrate the problem well: Akkermansia muciniphila predicts clinical response to PD-1 blockade in advanced NSCLC (73), yet its association with outcome is threshold- and context-dependent and has not been reproduced uniformly across cohorts (58, 74). Confounders that plausibly explain much of this inconsistency include geography and diet, ethnicity, tumour type, concomitant medication such as antibiotics, proton pump inhibitors and corticosteroids, and - importantly - confounding by indication, because patients who receive antibiotics are systematically sicker at baseline. Prospective studies should therefore report these variables and adjust for them rather than treating the microbiome as an isolated exposure.

### 6.2 Methodological barriers in microbiome profiling

The second barrier is methodological. Amplicon-based 16S rRNA gene sequencing detects only part of the community revealed by shotgun metagenomics, generally resolves taxa no further than genus level, and is subject to primer bias; in matched-sample comparisons performed on the same DNA, 16S sequencing identified fewer than half as many differentially abundant genera as shotgun sequencing, and every discordant result arose from failure of 16S to detect a genus rather than from a genuine biological difference (75). Wet-laboratory variability is at least as important: in a controlled comparison using mock communities and 139 positive controls, the DNA extraction method was the single largest contributor to variation in inferred composition, and barcode choice during library preparation also had a significant effect (76). Because 16S and shotgun workflows carry independent batch effects and there is still no consensus definition of a healthy microbiome, taxa that discriminate responders in one study can be created or erased by technical factors alone. Standardized reporting checklists and reference materials mitigate but do not eliminate this problem. Prospective microbiome-stratified trials should therefore pre-specify the sequencing platform and depth, a single extraction protocol, centralized bioinformatics, and pre-registered analysis plans.

### 6.3 Safety and regulatory barriers to fecal microbiota transplantation in immunocompromised patients

The third barrier is safety and regulation. FMT is not a benign intervention in immunocompromised hosts. In 2019, two patients enrolled in separate trials developed bacteraemia caused by extended-spectrum beta-lactamase-producing Escherichia coli transmitted through capsules manufactured from a single donor lot; whole-genome sequencing demonstrated clonal identity between donor and recipient isolates, and one recipient - a profoundly neutropenic allogeneic transplant recipient - died of sepsis (77). In 2020, the U.S. Food and Drug Administration reported six patients infected with enteropathogenic or Shiga toxin-producing E. coli after receiving stool-bank-manufactured FMT product, four of whom required hospitalisation, and two deaths were reported in patients who had received material from the implicated donor (79); the subsequent investigation showed that the Shiga toxin immunoassay used for donor screening has limited sensitivity in asymptomatic carriers (78). From a regulatory perspective, FMT is classified in the United States as both a biological product and a drug, so that use outside Clostridioides difficile infection requires an investigational new drug application, while classification within Europe remains fragmented between member states, ranging from a tissue or cell product in some countries to a medicinal product in others. For patients with NSCLC, most of whom are older and many of whom are lymphopenic after chemotherapy or thoracic radiotherapy, donor screening for multidrug-resistant organisms and Shiga toxin, restriction to well-characterized stool banks, and explicit informed consent are prerequisites rather than optional safeguards. An additional consideration specific to oncology is that donor-derived organisms may persist after transplantation, and the consequences of introducing a new, only partially characterized microbial community into a host who will subsequently receive further immunosuppressive therapy have not been quantified (38).

### 6.4 Ongoing clinical trials of microbial therapeutics in NSCLC

The fourth consideration is the design and scope of the evidence base itself. **Table 1** lists the interventional studies of microbial therapeutics that have been registered in NSCLC, together with their phase, population, intervention, status and primary endpoint. Three features of this landscape are striking. First, the field is dominated by small, predominantly single-arm studies: among the trials identified, only three randomise patients within an NSCLC-specific population, and these are small (15 to 100 patients) and use objective response or biomarker endpoints rather than survival endpoints. Second, no registry record in this population has posted results to its registry, and only one study - the phase 2 FMT-LUMINate trial, which met its primary endpoint with an objective response rate of 80% in first-line NSCLC - has been published in full (38). Third, the interventional landscape differs markedly by modality: FMT accounts for the largest share of trials and is the only modality that has been tested specifically in acquired resistance to PD-1 blockade, whereas single-strain probiotics and defined bacterial consortia are being tested predominantly in the first-line or adjuvant setting. Two further gaps are apparent. No registered trial in NSCLC has used a microbiome-modulating antibiotic strategy, a postbiotic, or a dietary intervention with a microbiome endpoint; and no trial of Clostridium butyricum with checkpoint blockade has been registered in this disease, so that the supportive evidence for this widely used probiotic remains retrospective (80). In addition, no randomised trial has yet used acquired resistance to PD-1 blockade as an entry criterion, so that the strategy with the greatest unmet clinical need still rests on single-arm evidence only (70, 71).

**Table 1:** Registered clinical trials of microbial therapeutics in non-small cell lung cancer.

Registry identifier	Phase	Population and intervention	Status	Primary endpoint
NCT04951583 (FMT-LUMINate)	2	Treatment-naive metastatic NSCLC with PD-L1 ≥50%; single healthy-donor FMT by oral capsule before pembrolizumab	Active, not recruiting	ORR; met in NSCLC (80%, 16/20) (38)
NCT06403111	2	First-line driver-gene-negative advanced NSCLC with PD-L1 <50%; FMT plus platinum doublet and tislelizumab	Recruiting	12-month PFS rate
NCT05669846	2	Relapsed or refractory PD-L1-positive NSCLC after anti-PD-(L)1 therapy; donor-derived FMT plus pembrolizumab	Recruiting	ORR (RECIST 1.1)
ChiCTR2000031411	Not stated	First-line EGFR/ALK wild-type metastatic non-squamous NSCLC; FMT plus pembrolizumab, pemetrexed and carboplatin; randomised 66 vs 34	Not stated	Not stated
ChiCTR2300076829	2	First-line advanced lung adenocarcinoma with PD-L1 expression 0; FMT plus PD-1 antibody and chemotherapy	Not stated	ORR; 6-month PFS rate
ChiCTR2100050952	No applicable	Treatment-naive EGFR/ALK-negative advanced NSCLC; Bifidobacterium lactis V9 versus placebo with ICI and platinum doublet; randomised, double-blind	Not stated	Microbiota, inflammatory and barrier endpoints in relation to efficacy
NCT04909034	Not applicable	Treatment-naive metastatic NSCLC with PD-L1 TPS ≥50%; MS-20 probiotic versus placebo with pembrolizumab; randomised, quadruple-masked	Completed	ORR at 48 weeks
ChiCTR2300070413	2	EGFR-mutant non-squamous NSCLC after EGFR-TKI failure; toripalimab, Clostridium butyricum and chemotherapy	Not stated	PFS
ChiCTR2300069317	4	Resected stage II-IIIA NSCLC with PD-L1 ≥1%; Clostridium butyricum with adjuvant atezolizumab	Not stated	Change in gut microbiota at 3 months
NCT03637803*	1/2	Advanced solid tumours including NSCLC progressing on PD-1/PD-L1 blockade; single-strain live biotherapeutic MRx0518 plus pembrolizumab	Terminated	Safety; clinical benefit
NCT05877430*	1/2	Advanced NSCLC (ICI-naive with PD-L1 ≥50%, or relapsed/refractory), head and neck cancer and melanoma; live biotherapeutic CJRB-101 plus pembrolizumab	Recruiting	Safety; efficacy
NCT05865730 (EV-2101)*	1/2	Stage IV non-squamous NSCLC with PD-L1 >50%, stable disease and Akkermansia-negative stool, plus clear-cell renal cancer; live Akkermansia muciniphila strain P2261 plus ICI	Recruiting	ORR at 9 months
NCT03686202 (MET-4)*	2/3	Advanced solid tumours including NSCLC receiving anti-PD-(L)1 monotherapy; defined faecal-microbiome consortium MET-4	Active, not recruiting	Changes in consortium strain abundance; treatment-related adverse events

Studies marked with an asterisk also enrol other tumour types. Registry entries that do not post a recruitment status are recorded as “not stated”. FMT, fecal microbiota transplantation; ICI, immune checkpoint inhibitor; ORR, objective response rate; PFS, progression-free survival; TPS, tumour proportion score.

### 6.5 Roadblocks to phase III standardization

Taken together, these observations define the roadblocks that stand between the current evidence base and a phase III trial, which are summarised with proposed mitigations in **Table 2**. The most fundamental is the absence of a validated predictive biomarker for patient selection. No microbial feature has been qualified for this purpose, there is no consensus on which measurement to use, and the same taxon can be associated with benefit and with harm in different cohorts (58, 73, 74, 82). The second is endpoint and design heterogeneity: phase II studies in this field have almost uniformly been single-arm and have used objective response as the primary endpoint, which cannot support registration, whereas a phase III programme will require a randomised, blinded, placebo- or sham-controlled design with a survival endpoint and pre-specified stratification by the microbial variable of interest. Feasibility of a randomised microbiome intervention in oncology has been demonstrated, but the operational requirements - blinding an intervention that alters stool composition, central product manufacture, and site-level harmonisation of sample handling - are substantial. The third roadblock is product standardisation. Donor selection criteria for FMT have been defined by a European consensus conference but are not oncology-specific (84), and there is no agreed potency or viability assay, no agreed delivery route, and no agreed lot-release specification; equivalent expectations for live biotherapeutic products are still evolving (86). The fourth is the regulatory pathway: only two faecal-microbiota-derived products have been licensed anywhere, both for recurrent Clostridioides difficile infection, so oncology has no approval precedent, and the classification of FMT differs between jurisdictions. The fifth is confounding, because antibiotics, proton pump inhibitors, corticosteroids, diet and geography all modify both the microbiome and the outcome; unless these are measured and adjusted for, a phase III result will be uninterpretable.

**Table 2:** Roadblocks to phase III standardization of microbial therapeutics in NSCLC, and proposed mitigations.

Roadblock	Why it prevents standardization for phase III	Proposed mitigation
No validated predictive biomarker	No microbial feature has been qualified for patient selection, there is no consensus on which measurement to use, and the same taxon is associated with benefit in some cohorts and with harm in others (58, 73, 74, 82)	Pre-specify a single primary microbial endpoint; move from taxonomy to functional and ecological readouts; qualify candidate scores prospectively in randomised cohorts
Endpoint and design heterogeneity	Phase II studies are almost uniformly single-arm with objective response as the primary endpoint, which cannot support registration; blinding an intervention that alters stool composition is operationally difficult	Agree a co-primary efficacy endpoint for phase III; use randomised, blinded, placebo- or sham-controlled designs with pre-specified stratification by the microbial variable
Product standardisation	Donor criteria for FMT are not oncology-specific (84); potency, viability and lot-release specifications, delivery route and batch consistency remain undefined, and expectations for live biotherapeutic products are still evolving (86)	Define oncology-specific donor and product specifications; manufacture under GMP with lot-release potency assays; fix a single delivery route
Regulatory pathway	Only two faecal-microbiota-derived products are licensed, both for recurrent Clostridioides difficile infection, so oncology has no approval precedent, and the classification of FMT differs between jurisdictions	Engage regulators early; align product characterisation with existing live-biotherapeutic guidance; prefer a defined consortium with a stable, characterisable composition
Confounding and external validity	Antibiotics, proton pump inhibitors, corticosteroids, diet and geography modify both the microbiome and the outcome, so an unadjusted result is uninterpretable	Measure and adjust for these variables; pre-specify antibiotic and proton pump inhibitor stewardship; harmonise dietary assessment across sites
Measurement standardisation across centres	DNA extraction, primer choice, sequencing platform and depth, and batch effects can each create or erase a signal (75, 76)	Adopt reporting checklists (83) and reference material (85); use a single extraction protocol with centralised sequencing and bioinformatics
Safety in immunocompromised patients	Transmission of multidrug-resistant organisms and Shiga toxin-producing E. coli has caused bacteraemia and death (77–79), and long-term safety data are absent	Screen donors for multidrug-resistant organisms and Shiga toxin; restrict to characterised stool banks; embed independent safety monitoring with extended follow-up

E. coli, Escherichia coli; FMT, fecal microbiota transplantation; GMP, good manufacturing practice.

The sixth roadblock is measurement standardisation across centres, because DNA extraction, primer choice, sequencing platform and depth, and batch effects can each create or erase a signal (75, 76); reporting checklists (83) and a faecal reference material (85) now exist, but their use is not yet mandatory in oncology trials. The seventh is safety in immunocompromised patients, which requires donor screening for multidrug-resistant organisms and Shiga toxin, restriction to characterised stool banks, and long-term post-transplant surveillance (77–79). Each of these roadblocks has a tractable mitigation, but none can be resolved by any single study; they require an international consortium approach with pre-registered protocols, a centralised laboratory and bioinformatic pipeline, standardised donor and product specifications, and a shared endpoint framework. It is worth noting that no completed and published phase III trial of a microbial therapeutic has yet been reported in any cancer, and that the only registry record in oncology carrying a phase 2/3 designation enrolls an all-comer solid tumour population and uses microbiome and safety endpoints rather than survival endpoints.

## 7 Concluding remarks and perspectives

The paradigm of NSCLC management is fundamentally shifting from a tumor-centric perspective to a holistic approach that integrates the host superorganism, identifying the gut microbiota as a pivotal regulator of systemic antitumor immunity. While the gut-lung axis has been elucidated to modulate immune checkpoint inhibitor efficacy through metabolic reprogramming, immune cell trafficking, and barrier maintenance, the field is now transitioning from correlative observations to predictive precision, evidenced by the emergence of ecological topology scores and specific fungal signatures. To fully realize the clinical potential of this axis, future research must overcome the challenges of individual heterogeneity by prioritizing longitudinal multi-omics integration to decipher causal interdependencies; this will pave the way for a shift from generic interventions to precision strategies, such as engineered probiotics and synergistic combinations, ultimately establishing the microbiome as a controllable variable for personalized oncology. This transition will require, however, that the field address its reproducibility, methodological and safety problems as rigorously as it pursues mechanism: standardized sample handling and sequencing, pre-registered analytical plans, harmonised donor selection criteria, and prospective recording of pharmacological confounders such as proton pump inhibitors and corticosteroids. Only then can the ecological variables identified here be converted into selection criteria robust enough to guide the treatment of individual patients, and in particular of those whose disease has already progressed on PD-1 blockade.
